# Supplementary material for: Baseline Characterization of the Gut Microbiota of Field and Colony Populations of Phlebotomus tobbi and Preliminary Assessment of the Anti-Leishmanial Activity of Cultivable Bacteria
Source: Pathogens. 2026 Jun 23;15(7):658. doi: 10.3390/pathogens15070658 (PMC13416292; doi:10.3390/pathogens15070658)
Supplement: Supplementary file 1 [file pathogens-15-00658-s001.zip › pathogens-4357168-File S1/Taxa2ASV_Decomposer/Family/f__Staphylococcaceae/seqs.mltree.pdf]

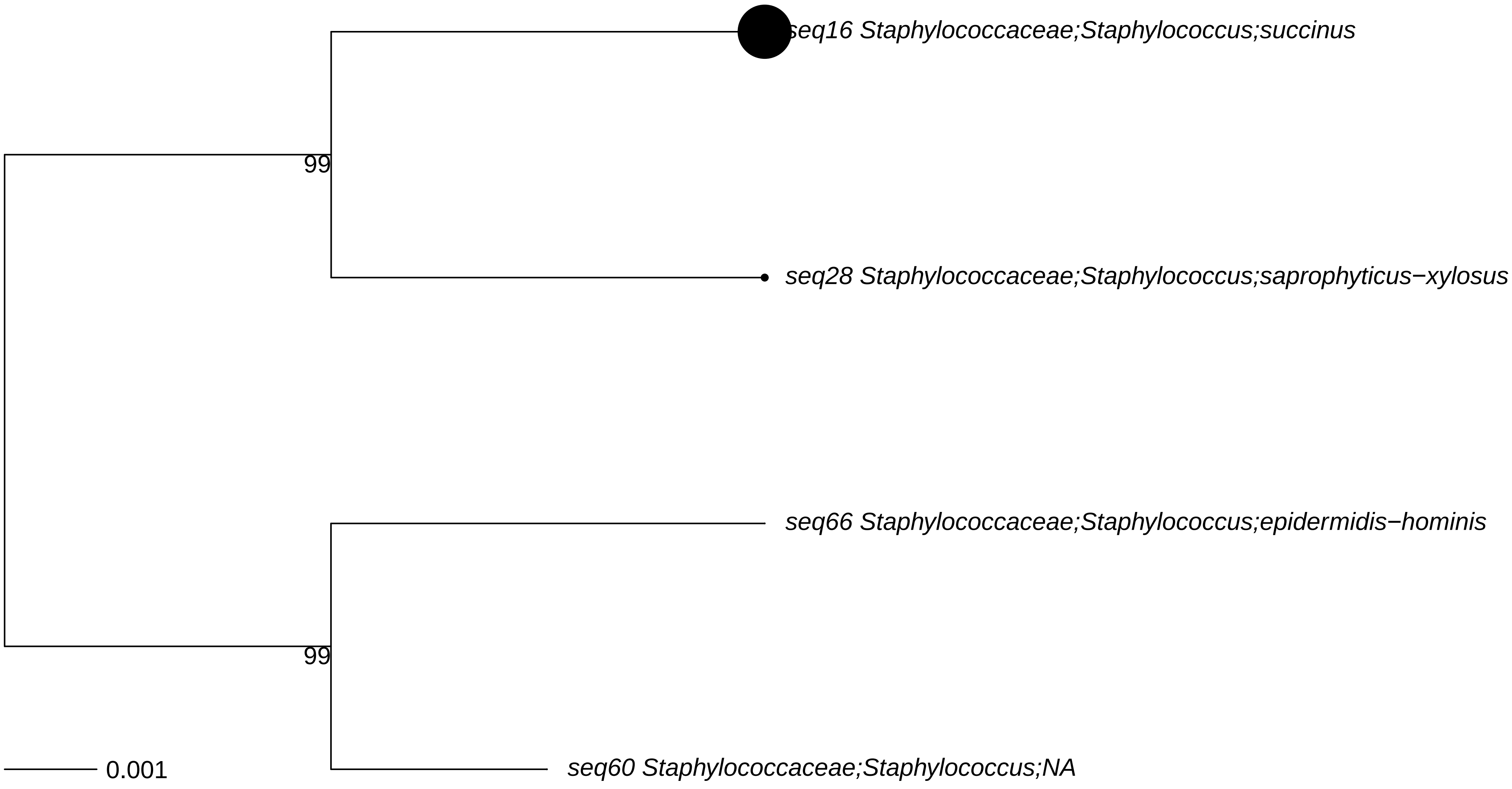
seq16 Staphylococcaceae;Staphylococcus;succinus
99
seq28 Staphylococcaceae;Staphylococcus;saprophyticus−xylosus
seq66 Staphylococcaceae;Staphylococcus;epidermidis−hominis
99
0.001
seq60 Staphylococcaceae;Staphylococcus;NA
